# Application of microcatheter shaping based on computational fluid dynamics simulation of cerebral blood flow in the intervention of posterior communicating aneurysm of the internal carotid artery

**DOI:** 10.3389/fneur.2023.1221686

**Published:** 2023-08-14

**Authors:** Gangqin Xu, Yueyang Ba, Kun Zhang, Dongyang Cai, Bowen Yang, Tongyuan Zhao, Jiangyu Xue

**Affiliations:** Cerebrovascular Division of Interventional Therapy Center, Zhengzhou University People's Hospital, Cerebrovascular Disease Hospital, Henan Provincial People's Hospital, Henan Provincial Neurointerventional Engineering Research Center, Zhengzhou, China

**Keywords:** aneurysm intervention embolization, intracranial aneurysm, internal carotid artery-posterior communicating artery aneurysm, microcatheter shaping, computational fluid dynamical

## Abstract

**Introduction:**

The present study aimed to investigate the application of the aneurysm embolization microcatheter plasticity method based on computational fluid dynamics (CFD) to simulate cerebral blood flow in the interventional treatment of posterior communicating aneurysms in the internal carotid artery and to evaluate its practicality and safety.

**Methods:**

A total of 20 patients with posterior internal carotid artery communicating aneurysms who used CFD to simulate cerebral flow lines from January 2020 to December 2022 in our hospital were analyzed. Microcatheter shaping and interventional embolization were performed according to the main cerebral flow lines, and the success rate, stability, and effect of the microcatheter being in place were analyzed.

**Results:**

Among the 20 patients, the microcatheters were all smoothly placed and the catheters were stable during the *in vitro* model test. In addition, the microcatheters were all smoothly placed during the operation, with a success rate of 100%. The catheter tips were stable and well-supported intraoperatively, and no catheter prolapse was registered. The aneurysm was completely embolized in 19 cases immediately after surgery, and a small amount of the aneurysm neck remained in one case. There were no intraoperative complications related to the embolization catheter operation.

**Conclusion:**

Microcatheter shaping based on CFD simulation of cerebral blood flow, with precise catheter shaping, leads to a high success rate in catheter placing, stability, and good support, and greatly reduces the difficulty of catheter shaping. This catheter-shaping method is worthy of further study and exploration.

## Introduction

Aneurysms of the posterior communicating artery (PComA) in the internal carotid artery (ICA) are common, accounting for approximately 25% of intracranial aneurysms (1). Interventional embolization is an important treatment modality for these types of aneurysms (2–4). Microcatheter shaping is the core technology of embolization, and good microcatheter shaping is beneficial for safe operation and satisfactory embolization effect (5, 6). However, due to the tortuous route of the intracranial segment of the internal carotid artery and the varying orientation of the aneurysm, the neck of the aneurysm is often accompanied by the posterior communicating artery. Conventional microcatheter “pigtail, C-shaped” shaping is often difficult to achieve or becomes unstable after being placed, and composite bending shaping is often required. Moreover, the specific shape is closely related to the surgeon’s personal experience, and young doctors have a longer learning cycle for catheter shaping. This study utilizes computational fluid dynamics to simulate the blood flow patterns of the host artery and related arteries of posterior communicating artery aneurysms. Based on the mainstream line, microcatheters are shaped to analyze their placement and stability during embolization. The aim is to explore a new catheter shaping method, reduce the difficulty of catheter shaping technology for posterior communicating artery aneurysms in the internal carotid artery, and improve the safety of surgery.

## Materials and methods

### Clinical data

A total of 20 patients were included in the present study, including 12 men and 8 women, aged 38–79 years, (mean 50 ± 6 years), with a total of 20 posterior communicating aneurysms of the internal carotid artery, all of which were unruptured cystic aneurysms presenting with narrow or relatively wide necks. The aneurysm sizes were approximately 2–8 mm. All of the cases were of the type where embolization alone was feasible to reduce the impact on the embolization catheter due to the use of stents, which affected the outcome judgment. The study was reviewed and approved by the Ethics Committee of the People’s Hospital of Zhengzhou University (approval number: 2019-085), and the patients or their families gave informed consent. Clinical data of patients including age, sex, underlying medical history, and imaging data were collected.

### Treatment method

#### Preoperative preparation

All patients underwent whole brain angiography and 3D-DSA of the parent artery before treatment to clarify the location, size, orientation, morphology, and relationship with the parent artery.

#### Establishment of CFD model

The DSA images of the 20 patients were obtained by the Siemens Artiszee Biplane VC14 DSA machine (Siemens, Germany) at the People’s Hospital of Zhengzhou University and exported in DICOM format. CFD raw data were acquired by referring to the method reported by Gao et al. (7), and the reconstructed images were repaired and removed by MeshLab (version 1.3.3) software to remove vascular fine branches. The images were exported in STL format to Harpoon software (version 4.3a) for 3D meshing. The fluid properties of blood and vascular boundary conditions were defined in Ansys 20.0 software, and the cardiac cycle for each hemodynamic calculation was divided into 800 steps with an average time of 0.001 s for each step without considering the differences in energy equations. The basic equation for simulating blood flow in the calculations was the Navier-Strokes equation. The vessel and aneurysm walls were set as rigid walls; the blood simulation was set as a Newtonian, incompressible, non-viscous fluid while ignoring the gravitational effect of blood. The blood flow pattern was set here as laminar flow, and the mean Reynolds coefficient was within the range of Reynolds coefficients of normal human intracranial vessels. The blood viscosity coefficient μ and blood density ρ were set to 0.00345 Pa and 1,050 kg/m3, respectively, and the inlet velocity was set to 0.32 m/s based on the measured average inlet velocity of healthy adult volunteers, and the pressure at the outlet was set to 0 Pa. By using Ensight software (version 9.0), the hemodynamic stress gradient of the aneurysmal lumen and parent arterial lumens could be quantified through color mapping. Additionally, blood flow streamlines within the spatial domain could be simulated. A three-dimensional model of the main blood flow in cerebral aneurysms could be established.

The spatial blood flow model formed by CFD simulation was further processed using Ansys 20.0 software and Ensight software (version 9.0), and the interactive visualization method was used to weaken the image of the vessel wall and enhance the intensity of the 3D hemodynamic flow map at the same time. The grayscale range was adjusted according to the color scale to preserve the fast main flow lines, while the slower peripheral flow lines were filtered out. The background vessel wall structure and the aneurysm part of the transparent and stereoscopic background were improved, and multi-angle projection was selected for retention analysis, for example, for the measurement of the degree of flow fold angle, segment length, and turning curvature, so that the 3D structure of the main blood flow into the aneurysm lumen with natural travel and fastest speed was more intuitively displayed and assembled.

#### Model preparation

All patient’s 3D-DSA data were obtained by Siemens Artiszee Biplane VC14 DSA machine (Siemens, Germany) and exported to DICOM format. After the repair and removal of fine branches of vessels by MeshLab (version 1.3.3) software and the creation of hollow models, they were imported into a 3D printer in STL format and printed with photosensitive resin using SLA (stereolithography apparatus) 3D printing technology, and the inner lumen of the model was 1:1 with the real lumen and wall thickness of 1 mm.

#### Microcatheter shaping

Headway-17 (Microvention, USA) shaping ratio is close to 1:1, so it was chosen as the shaping microcatheter. The shaping needle was inserted into the microcatheter according to the mainstream line for shaping. We performed steam fumigation on the microcatheter for 20s, and then it was put into cold water so that the shape could be set. The microcatheter was shaped, and then the *in vitro* model was carried out for the microcatheter in a place test to observe the microcatheter in place in the model. The position of the catheter, the position of the head end, and the support force of the catheter were observed.

#### Surgical procedure

After general anesthesia, the patient was routinely systemically heparinized, and a 6-guide catheter (an intermediate catheter was used for patients with vascular tortuosity) was introduced through the femoral artery approach and placed in the proximal skull base segment of the ipsilateral internal carotid artery. The optimal working angle was selected intraoperatively according to 3D-DSA reconstruction images. The head end of the microcatheter (Headway17) was shaped by 1:1 steam fumigation according to the main stream of cerebral blood flow (the length of the head end of the microcatheter was judged to be the length of the microcatheter that entered the aneurysm bend to a distance of two-thirds from the top of the aneurysm). According to the aneurysm size, micro-guide the wire with the embolization micro-catheter super-selected into the aneurysm lumen: (1) guide the wire to guide the catheter to the ocular segment of the internal carotid artery, slowly push the catheter and enter the aneurysm by itself; (2) guide the wire to guide the catheter to the distal end of the aneurysm, retract the micro-catheter, and pop the catheter into the aneurysm lumen; and (3) guide the wire into the aneurysm lumen, slowly follow up the catheter and enter the aneurysm lumen. The appropriate spring coil was selected for embolization, and dense embolization of the aneurysm was required to reduce recurrence (3, 8); wide carotid aneurysms were embolized using the stent semi-release technique. The imaging showed that the aneurysm was densely embolized and the embolization catheter was withdrawn.

### Postoperative follow-up

Patients were followed up clinically with modified Rankin Scale (mRS) scores at postoperative follow-up at 3, 6, and 12 months; imaging follow-up was performed by MRA or DSA from 6 to 12 months to observe aneurysm closure (Raymond embolization classification: grade I for complete aneurysm embolization, grade II for residual aneurysm neck, and grade III for partial aneurysm embolization).

## Results

During the treatment of 20 posterior communicating aneurysms in all patients, the microcatheters were smoothly placed in the microcatheter placement experiment in the *in vitro* model, and the catheter tip could be stabilized in different positions in the aneurysm lumen by pushing and pulling the microcatheter after the catheter was in place, and the head end was well pointed. Intraoperatively, 18 microcatheters were smoothly placed after one shaping, and in two patients, the catheter morphology changed due to heavy proximal vascular atherosclerosis and long operation time. In these cases, the catheter was shaped twice in the form of main blood flow and was then smoothly placed. Among the 20 cases, 18 were pushed into place, whereas in two cases, the guidewire was led to the distal end and retracted into place. The catheter was stable during embolization without dislodgement, none of the adjuvant techniques were used during the operation, and the aneurysm was completely embolized immediately after surgery in 19 cases. Only one case had a residual aneurysm neck. There were no intraoperative ruptures, bleeding, or other complications related to microcatheterization. All patients were followed up clinically for 3–6 months with an mRS0 score in seven cases. All 20 cases were followed up with imaging, showing complete embolization in 19 cases and a residual aneurysm neck in one case.

### Case

**Figure 1:**
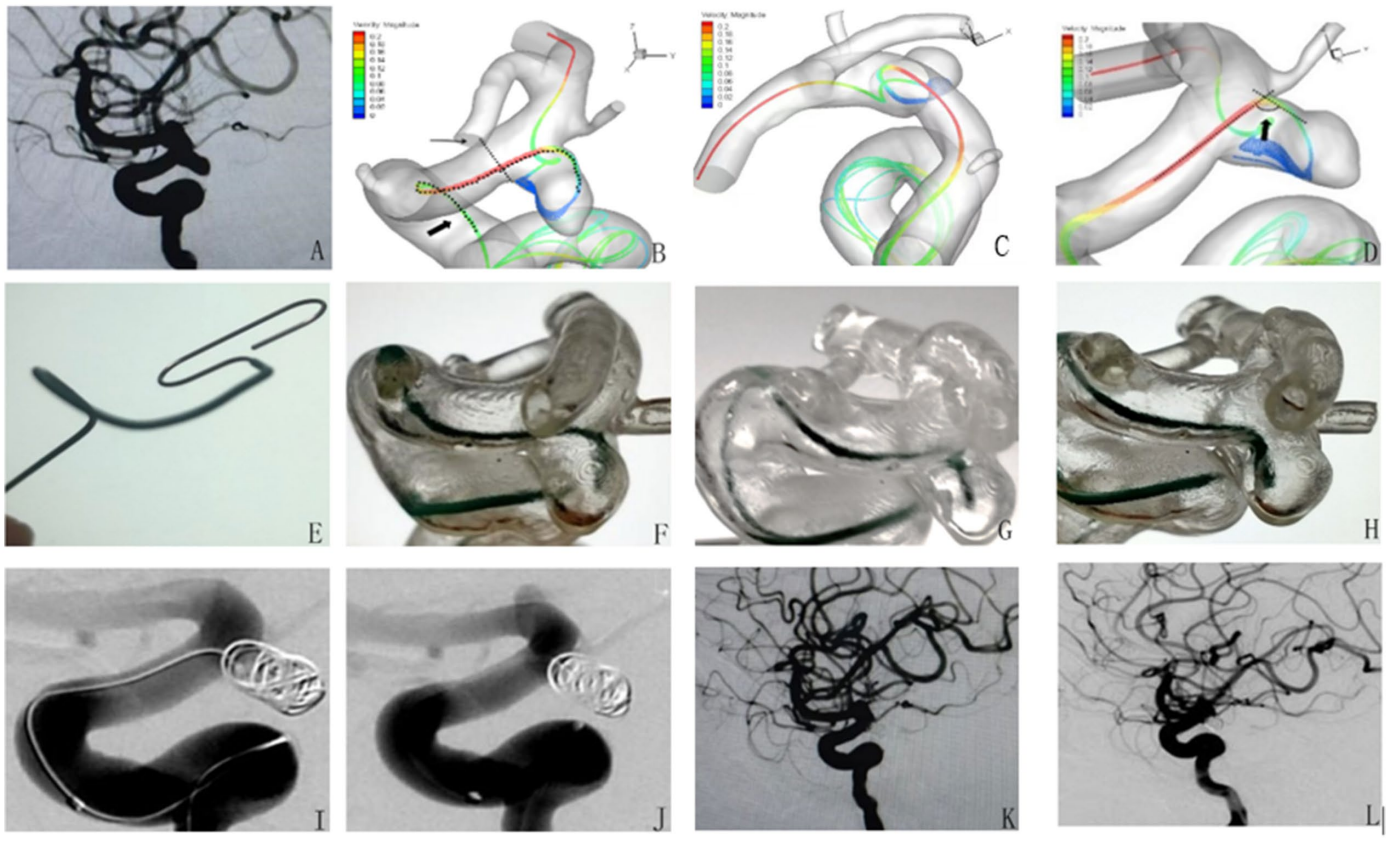
Application of microcatheter shaping based on computational fluid dynamics simulation of cerebral blood flow in the intervention of posterior communicating aneurysm of the internal carotid artery.

The patient is a 56-year-old woman admitted to the hospital with an intracranial aneurysm found on physical examination. Figure 1A shows a working position angiogram showing the right internal carotid artery posterior communicating aneurysm, size 4.1 mm*3.7 mm, relatively wide neck, aneurysm neck about 3.5 mm. Figures 1B–D show images of different angles of main blood flow 3D graphics. Figure 1E shows a 1:1 shaped Headway-17 microcatheter according to main blood flow 3D images. Figures 1F–H show the aneurysm model microcatheter being placed test: the microcatheter is smoothly placed, and then it is pushed and pulled so that the head end of the catheter can be stabilized in the distal end of the aneurysm neck (F), and the central part of the aneurysm neck (G) and the proximal end of the aneurysm neck (H) and the head end of the microcatheter are well oriented. Figures 1I,J show that the microcatheter was stable and well-supported during the intraoperative filling of the spring coil. Figure 1K shows that the aneurysm was densely embolized and the parent artery was patent on the immediate postoperative imaging. Figure 1L shows that the aneurysm healed well and the parent artery was patent on review 6 months after surgery.

## Discussion

Internal carotid artery-posterior communicating artery aneurysms are prone to growth and even rupture and bleeding due to their special anatomical location, and they have a high rate of disability and death (9). With the development of interventional techniques and the emergence of new devices, the efficacy and safety of interventional embolization have been improving, and embolization has gradually become one of the main treatments for aneurysms (6, 10). Microcatheter shaping for aneurysm embolization is the core technique of interventional procedures, and good microcatheter shaping can reduce the risk of intraoperative aneurysm rupture and increase the rate of complete aneurysm embolization to reduce recurrence (11, 12). Because of the tortuous course of the siphon segment of the internal carotid artery, the posterior communicating artery often emanates from the neck of the aneurysm, and the size, orientation, and morphology of the aneurysm varies, thus the operator needs to shape the microcatheter in various ways according to different characteristics. Traditional microcatheter shaping is mainly based on the operator’s own experience in shaping the microcatheter based on the 3D image of the vessel, which is highly subjective and subject to many imaging factors, and the accuracy of the catheter varies greatly among different levels of operators (6, 13, 14). Especially for posterior communicating aneurysms in which microcatheters are difficult to put in place, we often need to repeatedly make multiple shaping attempts and forceful guidance by microguide wires, which leads to repeated operations, long operation time, and even causes catastrophic consequences. Yamaguchi et al. (15) introduced a straight-tipped catheter into the parent artery, placed the head end near the aneurysm, left it in place for 5 min, relied on body temperature to shape the microcatheter according to the vessel morphology, and then removed the microcatheter and performed steam shaping of the last bend of the head end of the microcatheter into the aneurysm. This method reduces the number of bends in the shaping microcatheter by the operator, but the distal bend still requires shaping by the operator based on experience, which actually increases the surgical procedure time. The 3D printed aneurysm simulation model is helpful for the operator to have a more intuitive understanding of the aneurysm and the anatomy of the parent artery (16, 17), and some scholars have applied 3D printing technology to microcatheter shaping by printing a 1:1 vascular model based on the 3D image data of the aneurysm and performing microcatheter shaping based on the 3D model. The microcatheter is shaped according to the 3D model (18, 19), or the route of the microcatheter is artificially set inside the 3D image of the vessel using computer software, and then the morphological model of the route of the microcatheter is 3D printed and shaped accordingly. These techniques can enable the operator to understand the local anatomy of the aneurysm more intuitively so that the aneurysm embolization catheter shaping is more accurate. However, the nature of microcatheter shaping still requires the operator to rely on his or her own experience to plan the catheter route, as with traditional methods, and multiple shaping may be required to meet the surgical requirements. Some scholars 3D printed a 1:1 hollow model of the aneurysm, introduced the microcatheter into the model, placed the head end inside the aneurysm, thermoplasticized the whole, and then used the shaped microcatheter to perform surgery (20, 21). This method of shaping idea is different from the traditional way and reduces the reliance on the operator’s experience, but its straight microcatheter enters the aneurysm along the large curved side, and thus the head end of the microcatheter is not in the best position and it cannot solve the rebound problem during the use of the microcatheter after shaping (22). Hemodynamics plays a crucial role in the occurrence, development, and rupture of intracranial aneurysms. The traditional aneurysm shaping method completely relies on the operator’s surgical experience and does not consider the hemodynamic problems closely related to the aneurysm. The present study innovatively proposes a method of microcatheter shaping based on CFD simulation of cerebral blood flow. First, CFD simulation of cerebral blood flow is used to extract 3D images of the main blood flow. The microcatheter is shaped according to the 3D image, which is completely different from the traditional catheter shaping method, free from the dependence on surgical experience, and only requires 1:1 shaping of the microcatheter (the shape of the shaped microcatheter) according to the 3D mainstream line map. In this group of patients, the preoperative 3D printed hollow aneurysm model and *in vitro* model test visually observed that the microcatheter was smoothly placed and the position of the catheter tip in the aneurysm lumen was ideal during dynamic adjustment of the microcatheter, which confirmed the accuracy and stability of catheter shaping. The microcatheter was smoothly placed and the tip was well positioned when performing aneurysm embolization, and the tip of the catheter was well pointed and stable when filling the aneurysm, allowing for easy adjustment and a significant reduction in operative time and improved safety.

This study has some limitations: (1) clear 3D imaging data of the aneurysm need to be obtained before surgery, and the imaging effect of small branch aneurysms is often poor and thus difficult to use; (2) CFD flow simulation and generation of mainstream lines require a large amount of data processing, and some data need to be processed manually, which is time-consuming and not suitable for emergency surgery; (3) only patients with posterior internal carotid artery traffic aneurysms were studied in this study. Furthermore, the number of cases was small. Thus, a controlled study with multi-site aneurysms and large samples is needed to further verify the feasibility and safety of the proposed catheter shaping method. Intelligent microcatheter shaping will be the development direction of microcatheter shaping. This method provides a better idea for intelligent microcatheter shaping, and the development of one-stop application software may provide greater convenience for its clinical application.

In summary, microcatheter shaping based on CFD simulation of cerebral blood flow is a new idea of microcatheter shaping, which can reduce the difficulty of microcatheter shaping, make microcatheter shaping more accurate and stable, thus shortening the operation time and improve the success rate and safety of the operation. The superiority of this method needs to be confirmed by further studies.

## Data availability statement

The original contributions presented in the study are included in the article/supplementary material, further inquiries can be directed to the corresponding author.

## Ethics statement

The studies involving human participants were reviewed and approved by the Ethics Committee of the People’s Hospital of Zhengzhou University. The patients/participants provided their written informed consent to participate in this study.

## Author contributions

GX: design of methodology, creation of models, and presentation of the manuscript. YB: programming and software. KZ: collection of the research data. DC: visualization of results. BY: verification of the research. TZ: oversight for the research. JX: supervision and revision of the manuscript. All authors contributed to the article and approved the submitted version.

## Funding

Funding for this research was provided by the National Natural Science Foundation of China (Grant No. 81601583) and by the National keypoint research and invention program of the Thirteenth of China (Grant No. 2016YFC1300702).

## Conflict of interest

The authors declare that the research was conducted in the absence of any commercial or financial relationships that could be construed as a potential conflict of interest.

## Publisher’s note

All claims expressed in this article are solely those of the authors and do not necessarily represent those of their affiliated organizations, or those of the publisher, the editors and the reviewers. Any product that may be evaluated in this article, or claim that may be made by its manufacturer, is not guaranteed or endorsed by the publisher.
